# Effects of pre-harvest application of melatonin, 24-epibrassinolide, and methyl jasmonate on flavonoid content in blueberry fruit

**DOI:** 10.3389/fnut.2024.1495655

**Published:** 2024-12-23

**Authors:** Hui Zheng, Yong Yang, Sizheng Wu, Fan Jia, Jiani Jiang, Lin Yu, Guangmei Ou, Man Shu, Wei Qin

**Affiliations:** ^1^College of Horticulture, Xinjiang Agricultural University, Urumqi, China; ^2^School of Pharmacy, Hunan University of Chinese Medicine, Changsha, China; ^3^Resource Plant Research Institute, Hunan Botanical Garden, Changsha, China; ^4^College of Agriculture, Xinjiang Hetian College, Hetian, China

**Keywords:** flavonoids, blueberry fruit, pre-harvest application, melatonin, 24-epibrassinolide, methyl jasmonate

## Abstract

The application of plant growth regulators is an effective method to enhance flavonoid content in certain fruits; however, there is limited research comparing the effects of different plant growth regulators. This study evaluated the impact of pre-harvest application with melatonin, 24-epibrassinolide, and methyl jasmonate on flavonoid content in blueberry fruit. All three plant growth regulators increased the total polyphenol content, total flavonoid content, antioxidant capacities, and the activities of key enzymes involved in flavonoid biosynthesis, including flavone synthase, flavanone 3-hydroxylase, flavonol synthase, anthocyanidin synthase, and leucoanthocyanidin reductase. Among these, melatonin exhibited the most significant effect. Further comparative analyses showed that 0.5 mM melatonin was particularly effective in promoting the accumulation of flavonols, anthocyanins, and flavanones, as well as specific compounds such as avicularin, astragalin, morin, and reynoutrin in blueberry fruit, whereas 1.0 μM 24-epibrassinolide was more effective in enhancing the accumulation of flavones and flavanols, such as quercetin, leucocyanidin, phloretin, and epicatechin. In general, melatonin resulted in a greater enhancement of flavonoid content in blueberry fruit compared to 24-epibrassinolide and methyl jasmonate. This study highlights the distinct effects of these three plant growth regulators on flavonoid accumulation in blueberry fruit, providing valuable insights for the production of high-quality blueberries.

## 1 Introduction

Blueberry fruit, often referred to as the “king of the world fruit,” is renowned for its delicious taste and a rich array of healthy functional components, including plant polyphenols, organic acids, superoxide dismutase, dietary fibers, vitamins, and minerals (1). Among these components, flavonoids stand out for their strong antioxidant properties. Flavonoids are considered the primary contributors to the health benefits of blueberries, such as vision protection, cardiovascular improvement, anti-aging effects, enhanced immunity, and other functional benefits (2). Therefore, the flavonoid content, particularly anthocyanins, has become a key indicator for evaluating the nutritional quality of blueberry fruit (3).

The synthetic pathways of flavonoids in many plants have been extensively studied (4, 5). Flavonoids are synthesized through the phenylpropane pathway in the plant cytoplasm and are regulated by enzymes within this synthetic pathway (5). Key enzymes involved in the production of common flavonoid precursors include phenylalanine ammonia-lyase (PAL), cinnamate-4-hydroxylase (C4H), 4-coumarate-CoA ligase (4CL), chalcone synthase (CHS), and chalcone isomerase (CHI). Enzymes such as flavone synthase (FNS), flavanone 3-hydroxylase (F3H), flavonol synthase (FLS), leucoanthocyanidin reductase (LAR), and anthocyanidin synthase (ANS) are critical for producing the five major subclusters of flavonoid compounds (5).

The genes encoding these enzymes are structural genes within the flavonoid synthesis pathway.

During enzymatic flavonoid synthesis, the expression of these structural genes is regulated by interactions between different families of transcription factors. The MYB, bHLH, and WD40 are the three main transcription factors that regulate the structural genes of plant flavonoid synthesis. Moreover, these transcription factors often form MYB-bHLH-WD40 complexes to regulate structural gene expression directly (6).

Environmental factors such as light, temperature, water, and nutrients significantly affect the accumulation of flavonoids in plants. However, compared to methods such as modifying light or temperature conditions, applying plant growth regulators is a more convenient and practical method to enhance flavonoid accumulation in plants.

In addition, compared to other pesticides, such as pest control agents, herbicides, and fungicides, the rational use of plant growth regulators is more beneficial for sustainable agricultural development and environmental protection (7, 8). Plant growth regulators, such as ethylene, abscisic acid, jasmonates, brassinosteroids, cytokinin, auxin, and gibberellin, have been shown to be involved in the synthesis of flavonoids in fruits (9). Among these, the application of exogenous brassinosteroids and jasmonates has consistently demonstrated positive regulatory effects (7).

Brassinosteroids are a group of polyhydroxylated sterol hormones that are widely found in plants. They regulate plant growth and developmental processes and provide protection against various abiotic and biotic stresses (10). Notably, exogenous brassinosteroid treatment can increase the content of flavonoids in apples (11), strawberries (12), and pomegranates (13). Li et al. also reported that 24-epibrassinolide (EBL) increased the expression levels of PAL, CHI, flavonoid 3′5’-hydroxylase (F3’5’H), and LAR and induced the considerable accumulation of flavonoids in grapes (14). The jasmonates include methyl jasmonate (MeJA), jasmonic acid, and related oxylipins (9). Exogenous jasmonates have been used in crops to enhance crop stress resistance, improve fruit color, prolong fruit storage period, and promote plant growth (15). It has also been reported that jasmonates can improve flavonoid accumulation in certain fruits, such as pomegranates (16) and grapes (17). Ryu et al. found that under UV-B exposure, exogenous MeJA led to the upregulation of MdMYB10, MdCHS, and MdF3H genes, thus increasing flavonoid synthesis in apples (18). Melatonin (MT) was first reported in horticultural plants in 1995. Since then, the physiological functions of MT in plants have attracted increasing attention (8). MT is involved in plant growth and development stages, such as photosynthesis and biomass production, and protects plants against abiotic or biological stresses (19, 20). Therefore, MT is considered a new plant growth regulator. Recently, studies have shown that exogenous MT can also increase the accumulation of polyphenols in certain fruits, such as flavonoids in grape berries (21) and total phenols and anthocyanins in sweet cherry fruit (22). Xia et al. also demonstrated that exogenous MT application on grape berries increased the transcript abundance of anthocyanin synthesis-related genes and transcription factors MYBA1 and MYBA2 (23). The application of exogenous plant growth regulators has gradually increased fruit production in recent years. The physiological changes of flavonoids regulated by exogenous plant growth regulators in certain fruits are relatively clear, but many molecular regulation mechanisms have not been clarified. The regulation of plant growth regulators on the synthesis of fruit flavonoids is species-specific and tissue-specific (7). Different plant growth regulators have different effects on flavonoid synthesis in the same fruit, so the targeted and judicious selection of plant growth regulators is essential for effectively controlling and enhancing fruit quality.

At present, little work has been done to investigate the effects of pre-harvest spraying of different plant growth regulators on the nutrient content of blueberry fruit. This study investigated the effects of MT, EBL, and MeJA on conventional fruit quality, phenolic content, antioxidant capacity, enzyme activity, and flavonoid metabolites of blueberry fruit. The results of this study may provide useful information on the role of plant growth regulators on blueberry fruit growth and lay the foundation for producing high-quality blueberry fruit.

## 2 Materials and methods

### 2.1 Reagents

MT, EBL, MeJA, and Trolox were purchased from Shanghai Yuan Ye Biological Technology Co., Ltd. (Shanghai, China). Rutin and gallic acid were purchased from Chengdu Aifa Biotechnology Co., Ltd. (Chengdu, China). DPPH, ABTS, and FRAP were purchased from Shanghai Macklin Biochemical Technology Co., Ltd. (Shanghai, China). The ELISA Detection Kits of FNS, F3H, flavonol synthase (FLS), ANS, and leucoanthocyanidin reductase (LAR) were purchased from Jiangsu Meimian Industrial Co., Ltd. (Jiangsu, China). LC–MS-grade acetonitrile and methanol were purchased from Merck (Darmstadt, Germany). The remaining reagents were purchased from Sinopharm Chemical Reagent Co., Ltd. (Shanghai, China).

### 2.2 Spraying of plant growth regulators

The experimental site of this study was selected in the blueberry planting base of the Hunan arboretum (Changsha, Hunan, China), and the highbush blueberry ʻV3’ plants of uniform strength and age were selected. This study employed a completely randomized block design, with seven treatments and three fruit trees per treatment. These treatments comprised the application of 0.05, 0.5 mM MT, 0.1, 1.0 μM EBL, 0.5, and 5.0 mM MeJA, as well as a control treatment. The doses of the three plant growth regulators were determined based on related studies (23–25). The plant growth regulators were dissolved in distilled water containing 1% (v/v) ethanol and 0.2% (v/v) Tween 20, respectively. When most of the blueberry fruit was in the green mature stage, and the tops of the fruits were about to start turning red (25), the solutions were applied to the fruit clusters using small sprayers, ensuring complete coverage of all blueberry surfaces. Approximately 250 mL of solution was sprayed on each fruit tree. A water solution containing 1% (v/v) ethanol and 0.2% (v/v) Tween 20 was sprayed on the fruit clusters as the control treatment. All treatments were performed after sunset to avoid light decomposition of MT, EBL, and MeJA. When the blueberry fruit reached maturity, 40 blueberry fruit samples of the same size and color were randomly picked from the same direction of the three plants in each treatment. Thereafter, 15 of them were tested for conventional quality evaluation indicators on the day of picking, 25 of which were frozen in liquid nitrogen and stored in an ultra-low refrigerator freezer at −80°C for subsequent analysis.

### 2.3 Determination of fruit conventional quality evaluation

The fruit weight was weighed using an analytical balance with an accuracy of 0.001 g. The fruit’s transverse diameter and longitudinal diameter were measured with a vernier caliper. The TMS-Pro texture analyzer (Beijing Yingsheng Hengtai Technology Co., Ltd., Beijing, China) measured fruit firmness on the side of the fruit using a probe with a flat diameter of 2 mm. The puncture rate and the puncture depth were set to 1.0 mm/s and 5 mm, respectively. The maximum peak force during puncture was recorded as the fruit firmness, and the results were expressed in Newtons (N). The color of the fruit was measured from the side of the fruit by the CS-820 N desktop color photometer (Hangzhou Color Spectrum Technology Co., LTD, Hangzhou, Zhejiang, China) twice for each fruit, and the color was represented by the CEL L^∗^, a^∗^, and b^∗^values, where L^∗^represents the brightness (0 means black, 100 means white); a^∗^ represents the redness and greenness (a^∗^ > 0 means the degree of red; a^∗^ < 0 means the degree of green); b^∗^ represents yellowness and blueness (b^∗^ > 0 means the degree of yellow, b^∗^ < 0 means the degree of blue). A sample (5 g) was thoroughly ground in 30 mL of distilled water. Then, 10 mL of the mixture was diluted with distilled water to 100 mL and titrated with 0.1 mol/L NaOH to determine the fruit’s titratable acidity (TA). The result of TA was expressed as milligrams of citric acid per gram of fresh weight. The total soluble solids content (TSS) of the fruit was measured from fruit juice with the WAY-2 s Abbe refraction meter (Shanghai Zhuoguang Instrument Technology Co., Ltd., Shanghai, China) at 20°C, and the result was reported as ^°^Brix.

### 2.4 Determination of total polyphenol, total flavonoid, and total anthocyanin contents

The phenolic compounds in blueberry fruit were extracted according to the method of Karppinen et al. (26), with some modifications. The blueberry fruit powder (0.3 g), after grinding in the presence of liquid nitrogen conditions, was mixed with 3 mL of 70% ethanol containing 1% (v/v) hydrochloric acid and treated ultrasonically at 30°C for 50 min in the dark, then centrifuged at 10,000 rpm for 5 min. The supernatant was filtered through a 0.45 mm membrane, which was used to determine the total polyphenol content (TPC), total flavonoid content (TFC), total anthocyanin content (TAC), and antioxidant capacities. TPC was measured using the Folin–Ciocalteu (F–C) method (27). Briefly, 100 μl of extract solution was mixed with 7.9 ml of distilled water and 500 μl of Folin–Ciocalteu reagent, followed by the addition of 1.5 ml of 20% (w/v) Na_2_CO_3_ solution after 5 min. After incubating for 2 h at room temperature in the dark, the absorbance at 765 nm was measured using the UV-1780 spectrophotometer (Suzhou Shimadzu Instrument Co., Ltd., Suzhou, Jiangsu, China). The result was expressed as milligrams of gallic acid (GA) equivalent per gram of fresh weight of the sample (mg GA eq/g FW). TFC was determined using the NaNO_2_-Al (NO_3_)_3_ method (28). Briefly, 200 μl of extract solution was mixed with 400 μl of 5% (w/v) NaNO_2_ solution. After incubating for 6 min at room temperature in the dark, the mixture was added with 400 μl of 10% (w/v) Al (NO_3_)_3_ solution. After incubating for 6 min, 4 ml of 4% (w/v) NaOH solution and 5 ml of distilled water were added and left to stand for 15 min. The absorbance at 510 nm was measured using the UV-1780 spectrophotometer (Suzhou Shimadzu Instrument Co., Ltd., Suzhou, Jiangsu, China). The result was expressed as milligrams of rutin (RU) equivalent per gram of fresh weight of the sample (mg RU eq/g FW). TAC was determined by the double pH differential method (14). Briefly, 2.5 ml of extract solution was added to 7.5 ml of sodium acetate buffer (pH 4.5) and potassium chloride buffer (pH 1), respectively.

The absorbance of the two solutions was then measured at two wavelengths (520 and 700 nm) using a UV-1780 spectrophotometer (Suzhou Shimadzu Instrument Co., Ltd., Suzhou, Jiangsu, China). The TAC content was then calculated and expressed as an equivalent amount of anthocyanin-3-glucoside.

### 2.5 Determination of antioxidant activities

The DPPH and ABTS free radical scavenging capacities, along with the ferric reducing antioxidant (FRAP) value, are commonly used to evaluate the antioxidant activities of raw materials (29). The antioxidant activities of blueberry fruit were determined using phenolic compound extracts prepared as described in Section 2.4.

The DPPH and ABTS free radical scavenging capacities were measured based on the methods described in our previous study (27). Briefly, 100 μl of extract solution was mixed with 3.5 ml of DPPH solution and left to stand for 20 min in the dark. The absorbance was then measured at 517 nm using a UV-1780 spectrophotometer (Suzhou Shimadzu Instrument Co., Ltd., Suzhou, Jiangsu, China).

For ABTS scavenging capacity, 50 μl of extract solution was mixed with 4.9 mL of ABTS solution (prepared to an absorbance of 0.70 ± 0.02 at 734 nm) and left to stand for 10 min in the dark. The absorbance at 734 nm was then measured using the UV-1780 spectrophotometer (Suzhou Shimadzu Instrument Co., Ltd., Suzhou, Jiangsu, China).

The FRAP assay was performed according to the method described by Geng et al. (30). Briefly, the FRAP reagent was freshly prepared by mixing 300 mM acetate buffer (pH 3.6), 10 mM 2,4,6-tri(2-pyridyl)-1,3,5-triazine (TPTZ), and 20 mM FeCl_3_ in a ratio of 10:1:1, v/v/v). Then, a 100 μl extract solution was mixed with 4.9 ml of FRAP reagent and incubated at 37°C for 10 min.

The absorbance at 593 nm was measured using the UV-1780 spectrophotometer (Suzhou Shimadzu Instrument Co., Ltd., Suzhou, Jiangsu, China). The antioxidant activity results were expressed as micromoles of trolox equivalents per gram of fresh sample weight (μmol trolox/g).

### 2.6 Determination of enzyme activities associated with flavonoid synthesis

After grinding in liquid nitrogen conditions, the blueberry fruit powder (1.0 g) was mixed with 9 mL PBS at pH 7.2, then treated with a homogenizer and centrifuged at 2000 r/min for 20 min. The supernatant was collected for enzyme activity assays. The activities of FNS, F3H, FLS, ANS, and LAR were determined by corresponding ELISA detection kits according to the instructions, and results were expressed as U/g. The steps are as follows: 50 μl of standard solutions with different concentrations were added to the standard wells of the microplate, while 50 μl of the test samples were added to the sample wells of the microplate. Then, 100 μl of detection antibody labeled with horseradish peroxidase was added to each well, and the well was sealed and incubated at 37°C for 60 min. The liquid in the well was poured out, and each well was filled with washing solution and left to stand for 1 min. The washing solution was then poured out, and the absorbent paper was used to dry it. This washing step was repeated 5 times. Substrates A and B, 50 μl each, were added to each well and incubated at 37°C in the dark for 15 min. Subsequently, 50 μl of stop solution was added to each well, and the optical density values for each well were measured at 450 nm within 15 min using the Varioskan LUK microplate reader (Thermo Fisher Scientific Inc., Shanghai, China). The standard curve was plotted through the standard solution concentrations and corresponding optical density values, and the enzyme activity of each sample was calculated according to the curve equation.

### 2.7 Widely targeted metabolome methods

#### 2.7.1 Sample preparation and extraction

Anthocyanins are the most significant compounds in ripe blueberry fruit. With TAC as the main evaluation indicator, blueberry fruits treated with the control, 0.5 mM MT, 1.0 μM EBL, and 5.0 mM MeJA were selected for further analyses of flavonoid compounds using a widely targeted metabolome technique. The blueberry fruit was freeze-dried and ground to obtain the sample powder. Then, sample powder (50 mg) was dissolved in 1.2 ml of 70% methanol aqueous internal standard extract pre-cooled at −20°C. The solution was rotated every 30 min for 30s each time, 6 times in total. The supernatant was obtained by centrifugation at 12,000 r/min for 3 min and then filtered by a 0.22 μm microporous filter membrane before being stored for further analyses.

#### 2.7.2 UPLC conditions

The UPLC-ESI-MS/MS system (UPLC, ExionLC™ AD) and tandem mass spectrometry system were utilized to analyze the sample extracts. HPLC conditions utilized a reverse phase column (Agilent SB-C18 1.8 μm, 2.1 mm × 100 mm) with a column temperature of 40°C, an injection volume of 4 μl, and a flow rate of 0.35 ml/min. The mobile phases were ultra-pure water (0.1% formic acid added) (A) and acetonitrile (0.1% formic acid added) (B). The program of gradient elution: 0.00 min, 5% B; 0.00–9.00 min, 5–95% B; 9.00–10.00 min, 95% B; 10.00–11.10 min, 95–5% B; 11.10–14.00 min, 5% B. The effluent was alternatively connected to an ESI-triple quadrupole-linear ion trap (QTRAP)-MS.

#### 2.7.3 ESI-Q TRAP-MS/MS conditions

Primary conditions for MS include maintaining the electrospray ionization (ESI) temperature at 550°C, setting the ion spray voltage (IS) to 5,500 V in positive ion mode and -4500 V in negative ion mode, and adjusting the ion source gas I, gas II, and curtain gas to 50, 60, and 25 psi, respectively. Additionally, high collision-induced ionization parameters were employed. The instrument was tuned and calibrated with 10 and 100 μM polypropylene glycol solutions in QQQ and LIT modes. The QQQ scan used an MRM mode and had the collision gas (nitrogen) set to medium. The declustering potential and collision energy were completed by further optimization of the declustering potential and collision energy of each MRM ion pair.

#### 2.7.4 Qualitative and quantitative analyses of metabolites

The substance was characterized based on the secondary spectrum information based on the MWDB database (MetWare Biological Science and Technology Co., Ltd., Wuhan, Hubei, China). Isotopic signals, repeated signals containing K^+^, Na^+^, and NH_4_^+^, as well as fragments of other larger molecular weight substances, were removed during the analysis. After the basic MS data of metabolites were obtained, the relative metabolite content in different samples was represented by chromatographic peak area.

The processed data have been transferred to R software^1^ for PCA, OPLS-DA, and hierarchical cluster analysis. Differential metabolites for two-group analysis were identified based on VIP (VIP > 1) and absolute FC (FC > 1.5), with VIP values extracted from the results of OPLS-DA. The identified metabolites were matched with the KEGG compound database and then linked to the KEGG pathway database. The pathways containing significantly regulated metabolites were analyzed using metabolite sets enrichment analysis, and their significance was determined using *p*-values from a hypergeometric test.

### 2.8 Statistical analysis

In addition to the samples of fruit weight, transverse diameter, longitudinal diameter, firmness, and color L^*^, a^*^, and b^*^ were prepared and analyzed in 10 copies, and samples of other indicators were prepared and analyzed in triplicate. The data were expressed as the means ± sd. Multiple group comparisons were conducted using ANOVA and Duncan multiple tests in IBM SPSS version 23 (*p* < 0.05).

## 3 Results

### 3.1 Fruit conventional quality evaluation

The effects of MT, EBL, and MeJA on conventional indicators of blueberry fruit are shown in Table 1. Compared to the control, spraying 0.05 and 0.5 mM MT had no significant effects (*p* > 0.05) on fruit weight, transverse diameter, longitudinal diameter, firmness, color L^*^, a^*^, b^*^, TA, and TSS of blueberry fruit. Compared to the control, spraying with 0.1 and 1.0 μM EBL had no significant effects (*p* > 0.05) on fruit weight, transverse diameter, longitudinal diameter, firmness, color L^*^, TA, and TSS of blueberry fruit. Compared to the control, spraying 0.5 and 5.0 mM MeJA had no significant effects (*p* > 0.05) on fruit weight, transverse diameter, longitudinal diameter, firmness, color L^*^, a^*^, and TA. Comparison between different doses of the same plant growth regulator showed no significant difference (*p* > 0.05) between 0.5 and 5.0 mM MT on the conventional indicators of blueberry fruit. Compared with 0.1 μM EBL, 1.0 μM EBL significantly (*p* < 0.05) decreased color a^*^ and b^*^ of blueberry fruit. Compared with 0.5 mM MeJA, 5.0 mM MeJA significantly (*p* < 0.05) decreased color b^*^ of blueberry fruit. The results showed that the type and spraying dose of plant growth regulators influenced the conventional indicators of blueberry fruit. However, overall, spraying MT, EBL, and MeJA at the experimental doses had minimal impact on these conventional indicators.

**Table 1:** Conventional quality indicators of blueberry fruit.

Samples	Control	MT (0.05 mM)	MT (0.5 mM)	EBL (0.1 μM)	EBL (1.0 μM)	MeJA (0.5 mM)	MeJA (5.0 mM)
Fruit weight (g)	1.37 ± 0.09	1.37 ± 0.22	1.30 ± 0.17	1.32 ± 0.11	1.30 ± 0.19	1.37 ± 0.18	1.35 ± 0.18
Transverse diameter (mm)	13.32 ± 0.77^ab^	12.81 ± 0.44^b^	12.99 ± 0.72^b^	13.39 ± 0.91^ab^	13.32 ± 0.75^ab^	13.91 ± 0.72^a^	13.24 ± 0.83^ab^
Longitudinal diameter (mm)	12.99 ± 0.83^ab^	12.27 ± 0.57^b^	12.62 ± 0.57^b^	12.83 ± 0.92^ab^	12.60 ± 0.70^b^	13.50 ± 0.73^a^	12.93 ± 0.81^ab^
Firmness (N)	1.61 ± 0.27	1.56 ± 0.24	1.58 ± 0.17	1.51 ± 0.15	1.57 ± 0.12	1.60 ± 0.13	1.56 ± 0.28
L^*^	34.20 ± 2.52	34.09 ± 2.82	33.53 ± 3.22	30.20 ± 1.49	32.09 ± 2.14	31.70 ± 2.31	33.31 ± 1.90
a^*^	−0.23 ± 0.47^bc^	0.06 ± 1.01^bc^	−0.17 ± 0.51^bc^	0.63 ± 0.59^a^	0.10 ± 0.54^b^	−0.23 ± 0.36^bc^	−0.33 ± 0.27^c^
b^*^	−5.02 ± 1.41^c^	−5.45 ± 1.41^c^	−5.35 ± 1.97^c^	−2.87 ± 0.98^a^	−4.03 ± 1.16^b^	−4.11 ± 1.17^b^	−5.47 ± 1.38^c^
TA (mg/g FW)	7.65 ± 0.88	7.17 ± 0.31	7.61 ± 1.16	7.35 ± 1.84	6.46 ± 1.24	8.27 ± 1.31	6.79 ± 0.54
TSS (^°^Brix)	11.93 ± 0.52^bc^	11.63 ± 0.68^c^	12.13 ± 0.65^bc^	11.80 ± 0.65^c^	12.07 ± 0.65^bc^	14.07 ± 0.38^a^	13.20 ± 0.14^ab^

All values were means ± sd, *n* = 10 (fruit weight, transverse diameter, longitudinal diameter, firmness, color L^*^, a^*^, b^*^); *n* = 3 (titratable acidity, TA; total soluble solids content, TSS). Color L^∗^ represents the brightness (0 means black, 100 means white); a^∗^ represents the redness and greenness (a^∗^ > 0 means the degree of red; a^∗^ < 0 means the degree of green); b^∗^ represents yellowness and blueness (b^∗^ > 0 means the degree of yellow, b^∗^ < 0 means the degree of blue). Values with different letters are significantly different in the same row (*p* < 0.05).

### 3.2 TPC, TFC, and TAC of blueberry fruit

**Figure 1:**
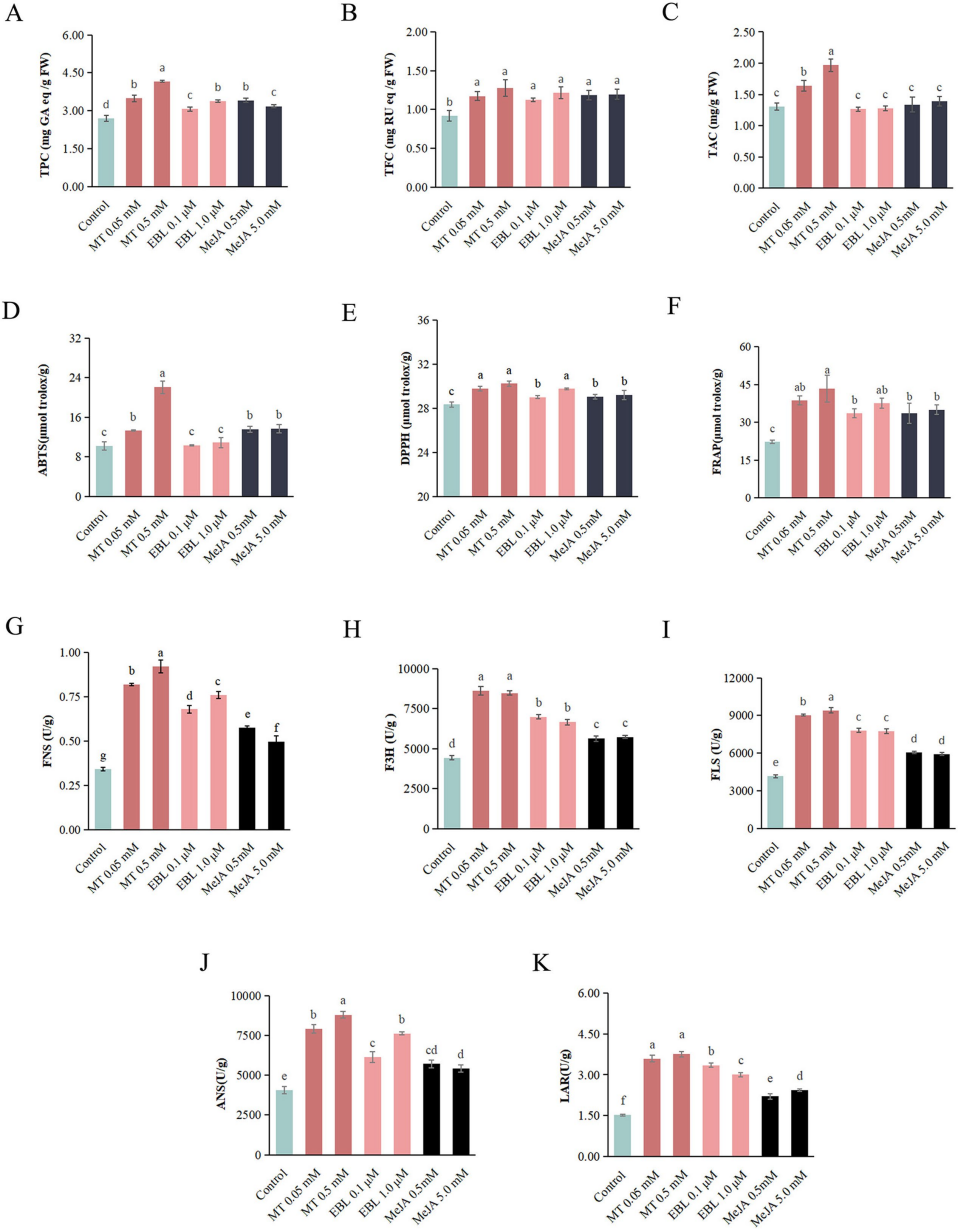
Phenolic compound contents, antioxidant activities and enzyme activities in blueberry fruit. **(A)** Total polyphenol content (TPC). **(B)** Total flavonoid content (TFC). **(C)** Total anthocyanin content (TAC). **(D)** ABTS free radical scavenging capacity. **(E)** DPPH free radical scavenging capacity. **(F)** Ferric reducing antioxidant power value (FRAP). **(G–K)** The activities of flavone synthetase (FNS), flavanone 3ẞ-hydroxylase (F3H), flavone synthase (FLS), anthocyanidin synthase (ANS), and leucoanthocyanidin reductase (LAR), respectively. Treatments followed by different letters are statistically different (*p* < 0.05).

The effects of MT, EBL, and MeJA on TPC, TFC, and TAC of blueberry fruit are shown in Figures 1A–C, respectively. Compared with the control, spraying MT, EBL, and MeJA all significantly increased (*p* < 0.05) the TPC and TFC of blueberry fruit. Among them, MT exhibited the most pronounced effect in enhancing TPC and TFC. The TPC of blueberry fruit sprayed with 0.5 mM MT was 1.54, 1.23, and 1.31 times higher than that of blueberry fruit sprayed with the control, 1.0 μM EBL, and 5.0 mM MeJA, respectively. The TFC of blueberry fruit sprayed with 0.5 mM MT was 1.39, 1.05, and 1.07 times higher than that of blueberry fruit sprayed with the control, 1.0 μM EBL, and 5.0 mM MeJA, respectively. Similar to TPC and TFC, MT increased the TAC of blueberry fruit better than EBL and MeJA. Compared to the control, MT significantly improved (*p* < 0.05) the TAC of blueberry fruit by 25.19 and 50.38% at 0.05 and 0.5 mM, respectively. However, spraying EBL and MeJA at experimental doses did not significantly change the TAC of blueberry fruit (*p* > 0.05) compared with the control. Comparison between different doses of the same plant growth regulator showed that 0.5 mM MT significantly increased (*p* < 0.05) the TPC and TAC of blueberry fruit more than 0.05 mM MT. Compared with 0.1 μM EBL, 1.0 μM EBL significantly increased (*p* < 0.05) the TPC of blueberry fruit. Compared with 0.5 mM MeJA, 5.0 mM MeJA significantly decreased (*p* < 0.05) the TPC of blueberry fruit. Overall, MT enhanced TPC, TFC, and TAC in blueberry fruit more effectively compared to EBL and MeJA at experimental doses, and the spraying doses of the same plant growth regulator also affected the phenolic compound contents of blueberry fruit.

### 3.3 Antioxidant activities of blueberry fruit

The effects of MT, EBL, and MeJA on the antioxidant activities of blueberry fruit, including the ABTS free radical scavenging capacity, DPPH free radical scavenging capacity, and FRAP value, are shown in Figures 1D–F, respectively. Compared with the control, spraying MT, EBL, and MeJA at experimental doses all increased the ABTS free radical scavenging capacity, DPPH free radical scavenging capacity, and FRAP value, and MT had a more substantial enhancing effect than EBL and MeJA. Compared with control, 0.5 mM MT significantly improved (*p* < 0.05) the ABTS free radical scavenging capacity, DPPH free radical scavenging capacity, and FRAP value of blueberry fruit by 116.49, 6.70, and 94.28%, respectively. Moreover, EBL and MeJA significantly increased (*p* < 0.05) the DPPH free radical scavenging capacity and FRAP value of blueberry fruit, with similar effects of both. The effect of MeJA on the ABTS free radical scavenging capacity of blueberry fruit was significantly higher (*p* < 0.05) than that of EBL. Comparison between different doses of the same plant growth regulator showed that 0.5 mM MT significantly increased (*p* < 0.05) ABTS free radical scavenging capacity of blueberry fruit compared with 0.05 mM MT, and 1.0 μM EBL significantly increased (*p* < 0.05) DPPH free radical scavenging capacity of blueberry fruit compared with 0.1 μM EBL. Additionally, there was no significant difference (*p* > 0.05) between 0.5 and 5.0 mM MeJA in promoting the three antioxidant capacities of blueberry fruit. The results indicated that MT, EBL, and MeJA enhanced the antioxidant capacity of blueberry fruit, with differing effects observed in different doses of the same plant growth regulator.

### 3.4 Enzyme activities associated with flavonoid biosynthesis of blueberry fruit

Spraying with MT, EBL, and MeJA may activate key enzymes involved in the flavonoid biosynthesis pathway of blueberry fruit. The five enzymes involved in the synthesis of five major subclusters of flavonoid compounds were focused on in this study. The activities of FNS, F3H, FLS, ANS, and LAR of blueberry fruit are shown in Figures 1G–K, respectively. Compared with control, 0.05, 0.5 mM MT, 0.1, 1.0 μM EBL, and 0.5, 5.0 mM MeJA all significantly increased (*p* < 0.05) FNS, F3H, FLS, ANS, and LAR activities of blueberry fruit. Of these, MT exhibited a stronger effect compared to EBL and MeJA. The FNS, F3H, FLS, ANS, and LAR activities of blueberry fruit sprayed with 0.5 mM MT increased by 171.18, 91.56, 126.79, 115.83, and 147.17% compared to control, respectively. Moreover, the promoting effects of EBL on the activities of FNS, F3H, FLS, and LAR of blueberry fruit were significantly higher (*p* < 0.05) than MeJA. A comparison between different doses of the same plant growth regulator showed that there was no significant difference (*p* > 0.05) in F3H and LAR activities of blueberry fruit sprayed with two doses of MT. However, FNS, FLS, and ANS activities of blueberry fruit sprayed with 0.5 mM MT were significantly higher (*p* < 0.05) than those of blueberry fruit sprayed with 0.05 mM MT. There was a significant difference (*p* < 0.05) in FNS, ANS, and LAR activities of blueberry fruit sprayed with two doses of EBL. There was also a significant difference (*p* < 0.05) in FNS and LAR activities of blueberry fruit sprayed with two doses of MeJA.

Overall, the results showed that MT, EBL, and MeJA enhanced the activities of the five flavonoid synthesis-related enzymes, with varying effects observed at different doses of the same plant growth regulator. Notably, these findings were consistent with the results of polyphenol compound contents.

### 3.5 Flavonoid metabolites of blueberry fruit

A widely targeted metabolome approach allows for a more comprehensive analysis of metabolite structures, enabling effective comparisons across specific metabolite categories and facilitating data mining (31).

**Figure 2:**
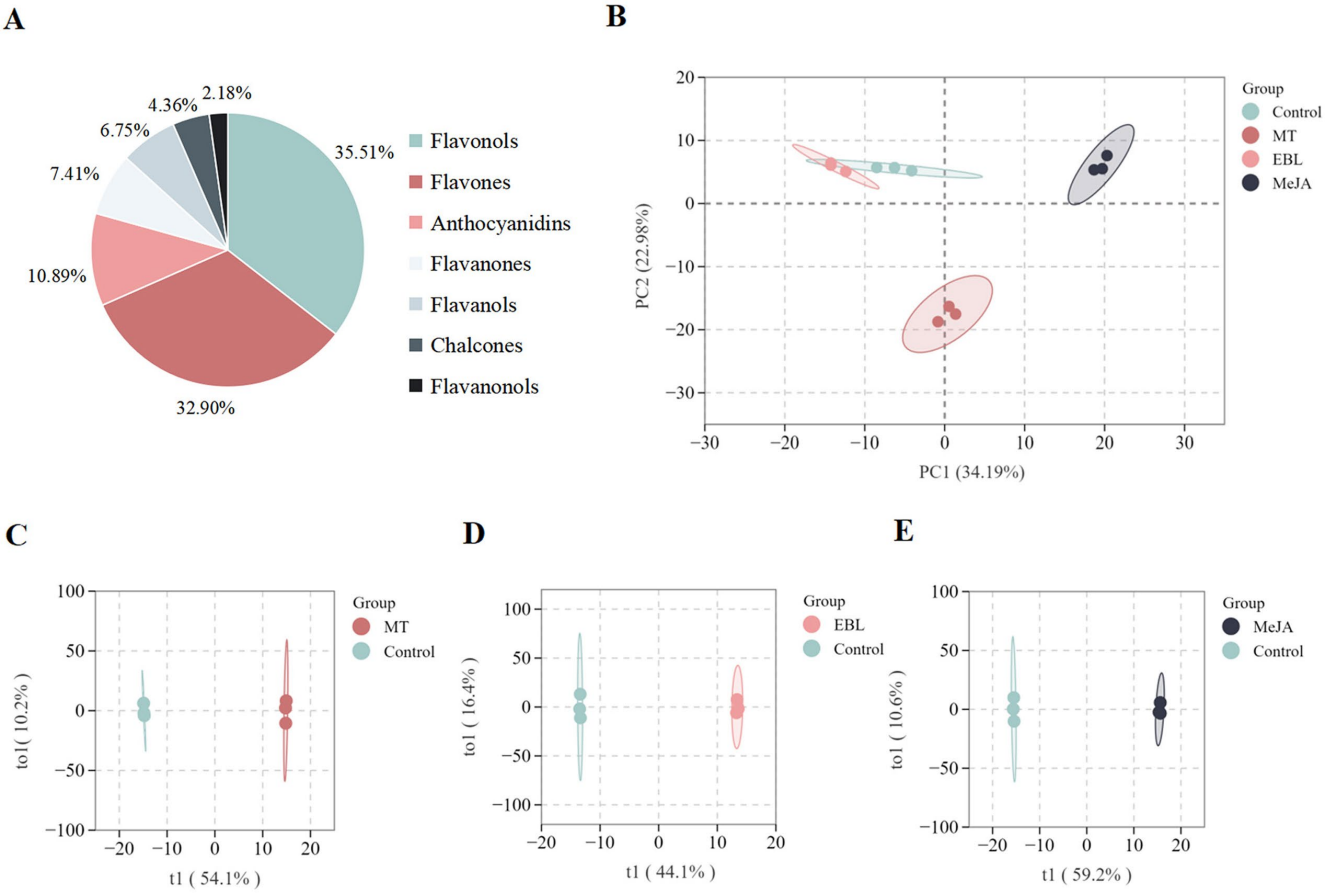
**(A)** Identified flavonoid metabolites in blueberry fruit. **(B)** PCA score plot of the four treatments. **(C–E)** Score plots of the OPLS-DA model for MT vs. control, EBL vs. control, and MeJA vs. control, respectively.

To further explore the effects of MT, EBL, and MeJA on flavonoid metabolites in blueberry fruit, a widely targeted metabolome analysis was conducted on four groups of blueberry fruit samples sprayed with 0.5 mM MT, 1.0 μM EBL, 5.0 mM MeJA, and control, respectively. As shown in Figure 2A, a total of 459 flavonoid metabolites were identified in blueberry fruit. These included 163 flavonols (35.51%), 151 flavones (32.90%), 50 anthocyanidins (10.89%), 34 flavanones (7.41%), 31 flavanols (6.75%), 20 chalcones (4.36%), and 10 flavanonols (2.18%). The PCA score plot of the flavonoid metabolites in blueberry fruit for the four groups is shown in Figure 2B. The first principal component (PC1) and the second principal component (PC2) explained 34.19 and 22.98% of the original dataset, respectively. In general, the 0.5 mM MT and 5.0 mM MeJA clusters were separated from the control cluster, indicating that 0.5 mM MT and 5.0 mM MeJA could significantly alter the flavonoid metabolite profile of blueberry fruit. The OPLS-DA model score plots for MT vs. control (R^2^X = 0.643, R^2^Y = 1, and Q^2^ = 0.961), EBL vs. control (R^2^X = 0.606, R^2^Y = 1, and Q^2^ = 0.914), and MeJA vs. control (R^2^X = 0.69, R^2^Y = 1, and Q^2^ = 0.973) are shown in Figures 2C–E, respectively. Q^2^ > 0.9 in these models indicated that these models all were excellent and stable, which can be used for subsequent differential metabolite screening using VIP analysis.

### 3.6 Differential flavonoid metabolite analysis between each plant growth regulator and control

**Figure 3:**
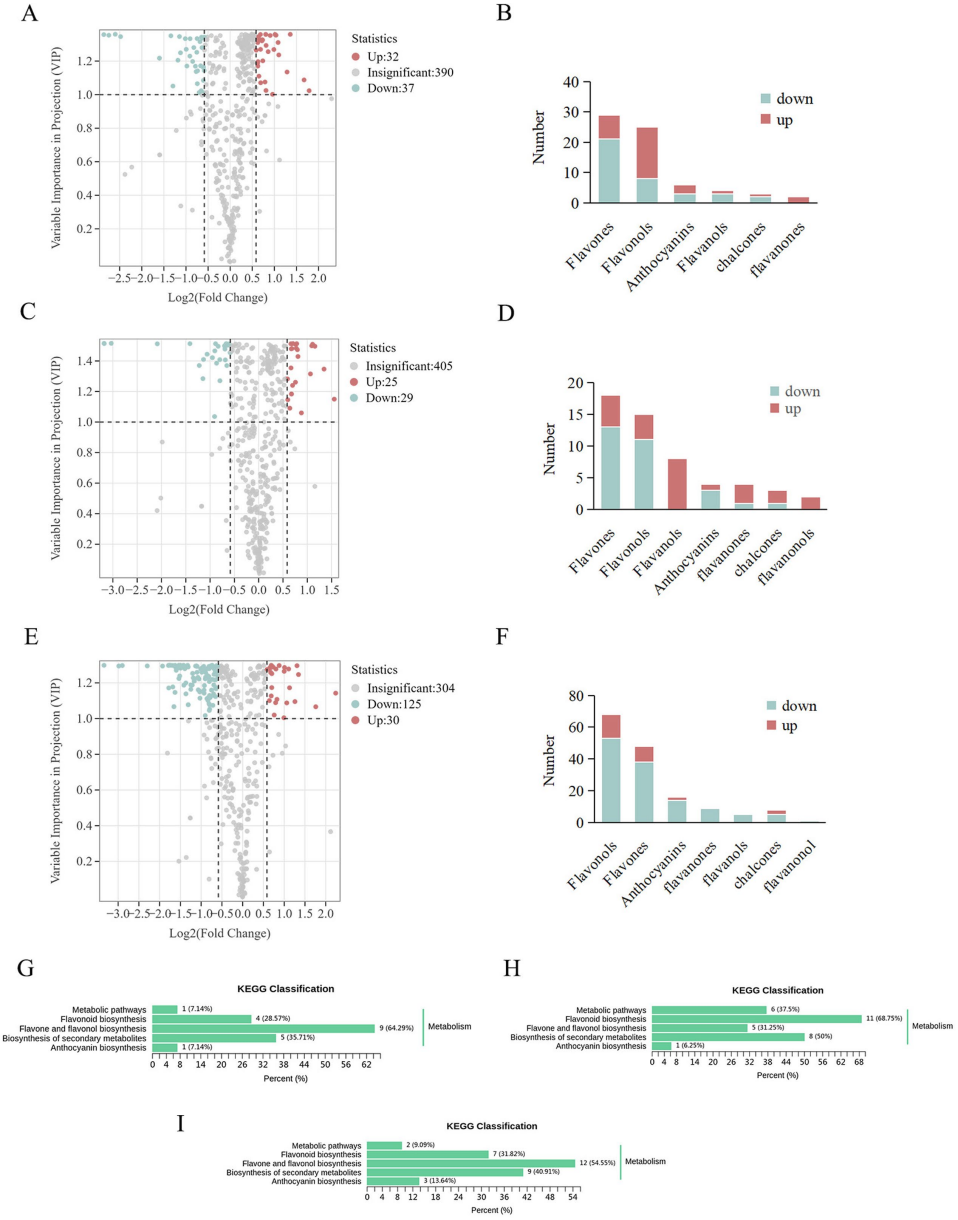
Volcano plots of the differential flavonoid metabolites (**A**: MT vs. control, **C**: EBL vs. control, **E**: MeJA vs. control). The differential flavonoid metabolite number (**B**: MT vs. control, **D**: EBL vs. control, **F**: MeJA vs. control). KEGG pathway analyses of the differential flavonoid metabolites (**G**: MT vs. control, **H**: EBL vs. control, **I**: MeJA vs. control).

The effects of each plant growth regulator on flavonoid metabolites in blueberry fruit were analyzed in comparison to the control. The volcanic plot of the differential flavonoid metabolites (VIP > 1, FC > 1.5) and the number of the differential flavonoid metabolites regulated in MT vs. control are shown in Figures 3A,B, respectively. There were 69 differential flavonoid metabolites, including 29 flavones (8 upregulated, 21 downregulated), 25 flavonols (17 upregulated, 8 downregulated), 6 anthocyanidins (3 upregulated, 3 downregulated), 4 flavanols (1 upregulated, 3 downregulated), and 3 chalcones (1 upregulated, 2 downregulated), and 2 flavanones (2 upregulated). The volcanic plot of the differential flavonoid metabolites (VIP > 1, FC > 1.5) and the number of the differential metabolites regulated in EBL vs. control are shown in Figures 3C,D, respectively. There were 54 differential flavonoid metabolites, including 18 flavones (5 upregulated, 13 downregulated), 15 flavonols (4 upregulated, 11 downregulated), 8 flavanols (8 upregulated), 4 anthocyanidins (1 upregulated, 3 downregulated), 4 flavanones (3 upregulated, 1 downregulated), 3 chalcones (2 upregulated, 1 downregulated), and 2 flavanonols (2 upregulated). The volcanic plot of differential flavonoid metabolites (VIP > 1, FC > 1.5) and the number of the differential metabolites regulated in MeJA vs. control are shown in Figures 3E,F, respectively. There were 155 differential flavonoid metabolites, including 68 flavonols (15 upregulated, 53 downregulated), 48 flavones (10 upregulated, 38 downregulated), 16 anthocyanidins (2 upregulated, 14 downregulated), 9 flavanones (9 downregulated), 8 chalcones (3 upregulated, 5 downregulated), 5 flavanols (5 downregulated), and 1 flavanonol (1 downregulated).

These results suggested distinct effects of MT, EBL, and MeJA on flavonoid subclusters in blueberry fruit.

According to the enrichment and annotation of the KEGG database, the differential flavonoid metabolites from three comparison groups (MT vs. control, EBL vs. control, and MeJA vs. control) were mainly related to ‘Flavonoid biosynthesis,’ ‘Flavone and flavonol biosynthesis,’ ‘Biosynthesis of secondary metabolites,’ and ‘Anthocyanin biosynthesis,’ as shown in Figures 3J–I. Flavones and flavonols were the two significant subclusters of flavonoids in blueberry fruit. MT vs. control, EBL vs. control, and MeJA vs. control were enriched 9/14 (64.29%), 5/16 (31.24%), and 12/22 (54.55%) in the ‘Flavone and flavonol biosynthesis’ pathway, respectively. Among them, MT vs. control and MeJA vs. control were significantly enriched. Anthocyanin is the key indicator for evaluating blueberry fruit’s color and nutritional quality. In this study, different flavonoid metabolites in the three comparison groups were also enriched in the ‘Anthocyanin biosynthesis’ pathway (MT vs. control, 1/14, 7.14%; EBL vs. control, 1/16, 6.25%; MeJA vs. control, 3/22, 13.64%).

### 3.7 Specific differential flavonoid metabolites among the three comparison groups

**Figure 4:**
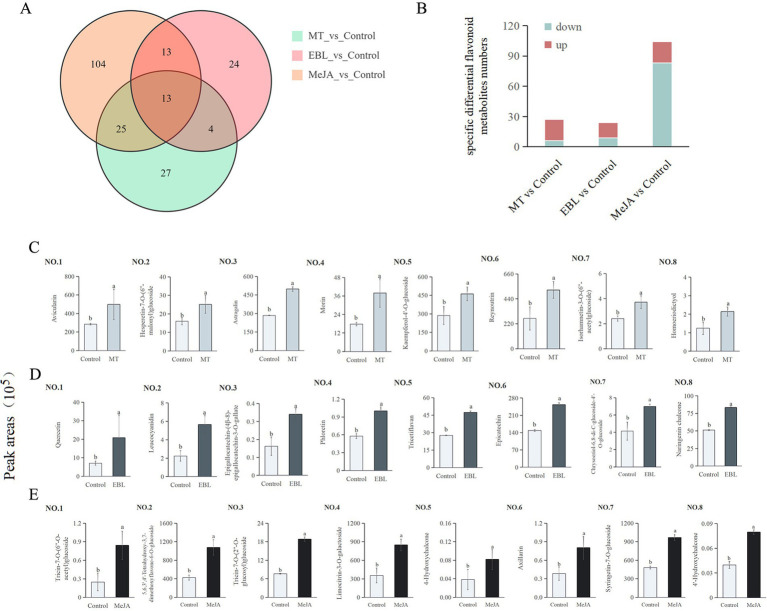
**(A)** Venn diagram showing the specific differential flavonoid metabolites among MT vs. control, EBL vs. control, and MeJA vs. control. **(B)** The specific differential flavonoids number. **(C–E)** Top 8 FC up-regulated specific differential flavonoid metabolites in MT vs. control, EBL vs. control, and MeJA vs. control, respectively. Treatments followed by different letters are statistically different (p < 0.05).

A total of 210 differential flavonoid metabolites were detected in the three comparison groups (Figure 4A), 13 of which were shared differential flavonoid metabolites. A total of 27, 24, and 104 specific differential flavonoid metabolites were identified in MT vs. control, EBL vs. control, and MeJA vs. control, respectively.

There were 27 specific differential flavonoid metabolites in MT vs. control, including 21 upregulated and 6 downregulated (Figure 4B). Among these upregulated specific flavonoids, the top 8 FC-specific differential flavonoid metabolites were avicularin, hesperetin-7-O-(6″-malonyl)glucoside, astragalin, morin, kaempferol-4’-O-glucoside, reynoutrin, isorhamnetin-3-O-(6″-acetylglucoside), and homoeriodictyol, as shown in Figure 4C 1–8. Hesperetin-7-O-(6″-malonyl)glucoside and homoeriodictyol belong to flavanones. The other 6 specific differential flavonoid metabolites belong to flavonols, which was consistent with the results in Figure 3B.

Compared to the control, the levels of avicularin, astragalin, morin, and reynoutrin in blueberry fruit treated with 0.5 mM MT increased by 75.38, 245.12, 114.35, and 94.35%, respectively. There were 24 specific differential flavonoid metabolites in EBL vs. control, including 15 upregulated and 9 downregulated (Figure 4B). Among these upregulated flavonoids, the top 8 FC-specific differential flavonoid metabolites were quercetin, leucocyanidin, epigallocatechin-(4β-8)-epigallocatechin-3-O-gallate, phloretin, tricetiflavan, epicatechin, chrysoeriol-6,8-di-C-glucoside-4’-O-glucoside, and naringenin chalcone, as shown in Figure 4D 1–8. Among them, 4 belong to flavonols, 2 to chalcones, 1 to flavonols, and 1 to flavones. Compared with control, the contents of quercetin, leucocyanidin, phloretin, and epicatechin in blueberry fruit sprayed with 1.0 μM EBL were increased by 193.74, 153.85, 73.55, and 69.87%, respectively. There were 104 specific differential flavonoid metabolites in MeJA vs. control, including 21 upregulated and 83 downregulated (Figure 4B). Among these upregulated flavonoids, the top 8 FC-specific differential flavonoid metabolites were tricin-7-O-(6”-O-acetyl)glucoside, 5,6,3′,4′-tetrahydroxy-3,7-dimethoxyflavone-6-O-glucoside, tricin-7-O-(2”-O-glucosyl)glucoside, limocitrin-3-O-galactoside, 4-hydroxychalcone, axillarin, syringetin-7-O-glucoside, and 4′-hydroxychalcone, as shown in Figure 4E 1–8. Among them, 3 belong to flavones, 3 to flavonols, and 2 to chalcones.

### 3.8 Difference in flavonoid subclusters

**Figure 5:**
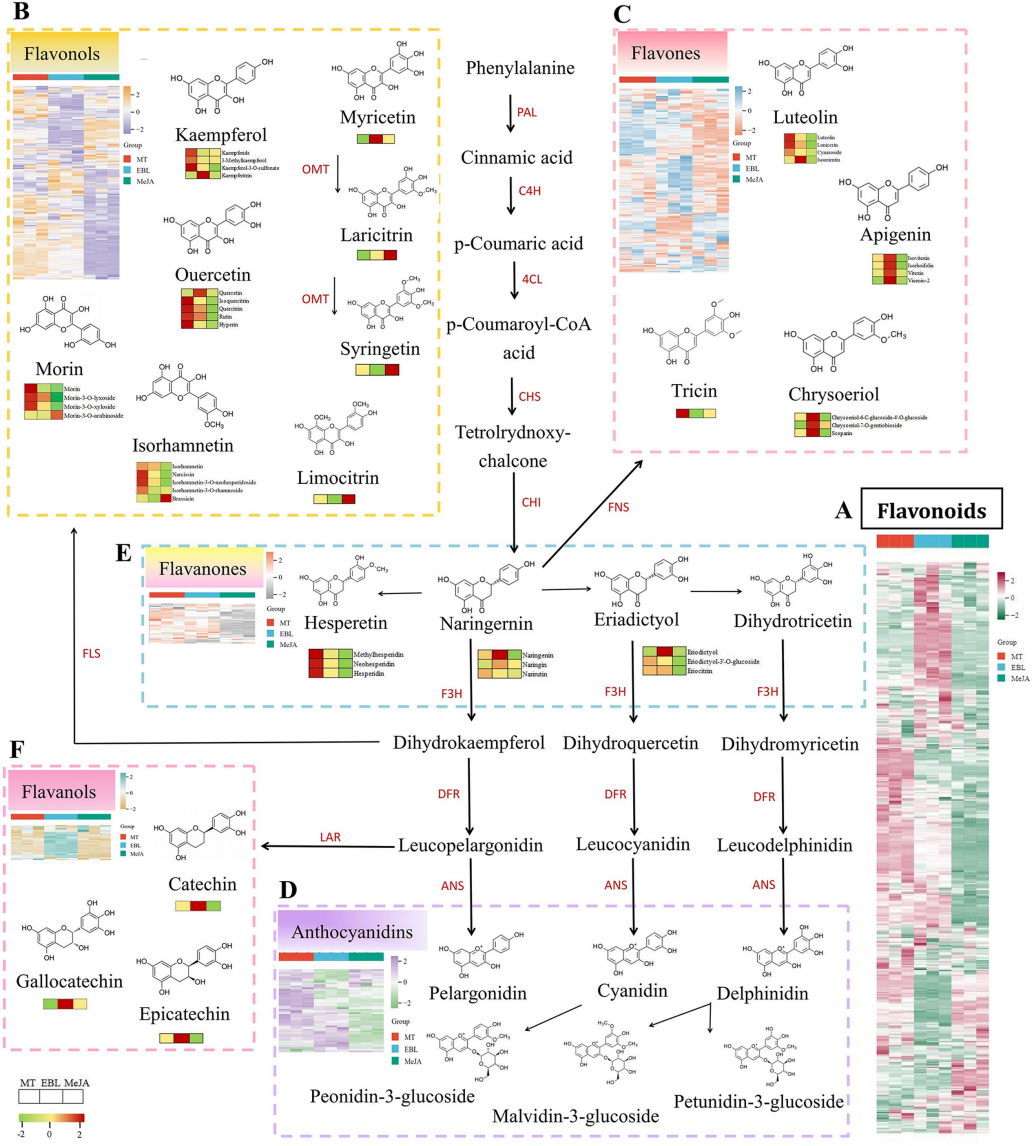
Heatmap of flavonoid subclusters and partially representative flavonoid metabolites, (**A**: Cluster of the total flavonoids, **B**: Cluster of the total flavonols, **C**: Cluster of the total flavones, **D**: Cluster of the total anthocyanidins, **E**: Cluster of the total flavanones, **F**: Cluster of the total flavanols), phenylalanine ammonia-lyase (PAL), cinnamate-4-hydroxylase (C4H), 4-coumarate-CoA ligase (4CL), chalcone synthase (CHS), chalcone isomerase (CHI), flavone synthase (FNS), flavanone 3-hydroxylase (F3H), flavonol synthase (FLS), O-methyltransferase (OMT), dihydroflavonol 4-reductase (DFR), leucoanthocyanidin reductase (LAR), and anthocyanidin synthase (ANS).

Hierarchical cluster analysis was utilized to visualize the relative abundance of metabolites, facilitating comparisons of the effects of MT, EBL, and MeJA on flavonoid subclusters in blueberry fruit. According to the cluster heatmap of total flavonoids (Figure 5A), blueberry fruit sprayed with MT had the highest abundance, followed by blueberry fruit sprayed with EBL and MeJA. The result was consistent with TPC, TFC, and enzyme activities. In addition, heatmap analyses were also conducted for the top 5 subclusters of flavonoids in blueberry, including flavonols, flavones, anthocyanins, flavanones, and flavanols (Figures 5B–F). Blueberry fruit sprayed with MT exhibited a higher relative abundance in flavonol, anthocyanin, and flavanone subclusters. Moreover, blueberry fruit sprayed with EBL showed a higher relative abundance in flavone and flavanol subclusters.

From the heatmap of total flavonols (Figure 5B), there were 99 compounds in two clusters with higher relative abundance in blueberry fruit sprayed with MT. This mainly included quercetin derivatives, kaempferol derivatives, isorhamnetin and its derivatives, and morin and its derivatives. There were 24 flavonol compounds in a cluster with a higher relative abundance in blueberry fruit sprayed with 1.0 μM EBL, mainly including kaempferol derivatives, quercetin, and its derivatives. In addition, 40 flavonol compounds were found in a cluster with a higher relative abundance in blueberry fruit sprayed with MeJA. Besides quercetin and kaempferol derivatives, they mainly include limo citrin, syringe, and lari citrin derivatives. Notably, the relative abundances of derivatives of laricitrin and syringetin were higher in blueberry fruit sprayed with MeJA than in blueberry fruit sprayed with MT and EBL. In this study, the flavones in blueberry fruit mainly included the derivatives of apigenin, luteolin, chrysoeriol, tricin, and diosmetin. From the heatmap of total flavones (Figure 5C), 54 compounds were found in a cluster with higher relative abundance in blueberry fruit sprayed with MT. This mainly includes apigenin derivatives, luteolin and its derivatives, and tricin derivatives. In addition, 49 flavone compounds were found in a cluster with higher relative abundances in blueberry fruit sprayed with EBL, mainly including apigenin derivatives and chrysoeriol derivatives.

Compared with MT and EBL, MeJA had no significant advantage in the accumulation of flavone compounds in blueberry fruit. Anthocyanins are the most important compounds for the color of blueberry fruit and an important nutritional indicator for the quality of blueberry fruit. From the heatmap of total anthocyanins (Figure 5D), six anthocyanins were found in blueberry fruit, including pelargonidin, cyanidin, peonidin, delphinidin, malvidin, and petunidin. The relative abundances of anthocyanin compounds in blueberry fruit sprayed with 0.5 mM MT were significantly higher than those sprayed with EBL and MeJA. These findings were consistent with the TAC and ANS activity results. There were relatively few flavanone and flavanol compounds in the flavonoids of blueberry fruit. The representative flavanol compounds of blueberry fruit were catechin, epicatechin, gallocatechin, and cinchonain. In the heatmap of total flavanones (Figure 5E), the relative abundances of blueberry fruit sprayed with MT were higher than those sprayed with EBL and MeJA, especially hesperetin and its derivatives. The relative abundances of flavanol compounds in blueberry fruit sprayed with EBL were higher than those sprayed with MT and MeJA, as shown in the heatmap of total flavanols (Figure 5F).

## 4 Discussion

Some plant growth regulators can be used as growth promoters by promoting cell division, supporting the growth and development of vegetative organs, preventing flowers and fruit drop, inducing fruit set, and accelerating ripening (32). Notably, these regulators promote the synthesis of flavonoids in certain fruits by speeding up fruit ripening, such as elevating apple anthocyanin response by ethylene induction (33) and enhancing sweet cherry anthocyanin production by auxin induction (34). This leads to the accumulation of flavonoids in fruit, which accelerates fruit aging and may reduce shelf life. Some plant growth regulators benefit from synthesizing flavonoids in fruits, not by accelerating fruit ripening, such as MeJA spray on apples (7, 35).

The regulation of plant growth regulators on flavonoid synthesis in fruits is both species-specific and tissue-specific (7). To assess the ripeness of blueberry fruit in this study, conventional quality indicators were measured. Overall, spraying MT, EBL, and MeJA at the experimental doses had minimal impact on the conventional indicators of blueberry fruit, particularly firmness and titratable acidity (TA), which are key evaluation criteria for fruit ripeness. Based on these findings, it was preliminarily concluded that these treatments did not significantly accelerate the ripening of blueberry fruit, suggesting that their application is feasible for the production of blueberry fruit.

Blueberries have long been considered health-promoting fruits due to their high levels of flavonoids and antioxidants (36). It has been shown that spraying MT (22), EBL (13), and MeJA (16) can increase TPC, TFC, and TAC in certain fruits, and similar results were obtained in this study. Furthermore, it was found that MT enhanced the phenolic compound contents of blueberry fruit more effectively than EBL and MeJA. Studies have also demonstrated that the antioxidant activity of anthocyanins was superior to that of flavonols and phenolic acids (37). Notably, MT promoted TAC in blueberry fruit better than EBL and MeJA, leading to higher antioxidant capacity of blueberry fruit sprayed with MT. As reported in Kalt’s study, anthocyanins are the main phenolic compounds in ripe blueberry fruit, comprising approximately 60% of TAC/TPC (38).

Due to different varieties and planting conditions, the TAC/TPC ratio in blueberry fruit ranged from 37 to 49% in this study. Among them, TAC/TPC in blueberry fruit sprayed with MT was higher than that sprayed with EBL and MeJA. The result may indicate that spraying MT is more effective in promoting the accumulation of anthocyanins in blueberry fruit compared with other flavonoid subclusters.

Flavonoids are synthesized by the phenylpropane pathway in plant cytoplasm and regulated by synthetic pathway enzymes. Previous studies have exhibited that the application of MT, EBL, and MeJA can effectively upregulate the expression of structural genes associated with flavonoid synthesis, thereby enhancing the activities of related enzymes in certain fruits (7, 11, 39). Therefore, the activities of FNS, F3H, FLS, ANS, and LAR of blueberry fruit were further investigated in this study. FNS catalyzes the synthesis of flavones from flavanones (40). F3H can catalyze the conversion of naringenin to dihydroflavonols, resulting in further biosynthesis of flavonols and anthocyanins in the later stage (41). FLS is a critical enzyme in forming flavonols and catalyzes the conversion of dihydroflavonols to flavonols (42). LAR and ANS can cause leucoanthocyanidins to convert to flavanols (2,3-trans-flavan-3-ols) and anthocyanins (3-OH-anthocyanins), respectively, indicating a competitive relationship between these pathways (43, 44). Consistent with the results of phenolic contents, MT, EBL, and MeJA all demonstrated a capacity to enhance the activities of FNS, F3H, FLS, ANS, and LAR of blueberry fruit in this study, among which MT had the best effect. In addition, the ANS activity of blueberry fruit sprayed with 0.1 μM EBL was significantly lower (*p* < 0.05) than that sprayed with 1.0 μM EBL, while the LAR activity of blueberry fruit sprayed with 0.1 μM EBL was significantly higher (*p* < 0.05) than that sprayed with 1.0 μM EBL. Similar results were also found in MeJA treatments sprayed with two doses. This may be because the LAR and ANS use the same substrate in the synthetic pathway, leading to a competitive relationship (43, 44).

Overall, enzyme activities associated with flavonoid synthesis may explain the differences in the accumulation of phenolic compounds in blueberry fruit induced by various plant growth regulators and doses in this study. The results of the widely targeted metabolome technique revealed that flavonols and flavones were the two major subclusters of flavonoid metabolites in blueberry fruit in this study, consistent with the findings of Zhang et al. (45). The result of the differential flavonoid metabolites of MT vs. control and the heatmaps of flavonoid subclusters both showed blueberry fruit sprayed with MT exhibited higher relative abundance in flavonol, anthocyanin, and flavanone subclusters. The results of the anthocyanin subcluster was consistent with those of TAC. Compared with EBL and MeJA, MT was beneficial in increasing the anthocyanin content in blueberry fruit. Although the activities of FNS, F3H, FLS, ANS, and LAR of blueberry fruit sprayed with MT were all higher than those in blueberry fruit sprayed with EBL and MeJA, MT may more effectively enhance the expression of FLS and ANS compared to FNS and LAR within the flavonoid synthesis pathway in blueberry fruit. Similar to Sun et al.’s study (46), spraying MT on red pear fruits during the pre-color-change period promoted FLS transcript levels and concentrations of anthocyanins and flavonols and decreased LAR transcript levels and concentrations of flavanols in fruit. Chen et al. (47) also reported that applying MT promoted the accumulation of anthocyanins, flavonols, and proanthocyanins and induced the expression of FLS, ANS, and LAR in crabapple leaves. In addition, compared to EBL and MeJA, MT significantly increased some notable compounds such as avicularin, astragalin, morin, and reynoutrin in blueberry fruit. Morin was initially isolated from the plant species of the Moraceae family and later found in the leaves, fruits, stems, and branches of many plants, with antioxidant, anti-tumor, anti-hypertensive, antibacterial, and other health benefits (48). In the analysis of flavonoids of 22 common fruits, Zhang et al. (45) found that the content of morin in blueberry fruit was the highest, and it has the potential to be a representative functional component of blueberry fruit. Avicularin, astragalin, reynoutrin, and homoeriodictyol are natural flavonoids with a wide range of physiological activities. Avicularin has been found to suppress inflammation in human chondrocytes (49). Astragalin exhibits anti-inflammatory effects (50).

Reynoutrin has shown the potential to improve ischemic heart failure (51). Homoeriodictyol demonstrates anticancer effects (52). Among the flavonoid subclusters in blueberry fruit, anthocyanins had the highest content and demonstrated remarkable antioxidant properties. Thus, they can serve as a key indicator for comparing the effects of MT, EBL, and MeJA. Based on the above analysis, MT appears to be more suitable than EBL and MeJA for producing high-quality blueberry fruit.

The result of the differential flavonoid metabolites of EBL vs. control and the heatmap of flavonoid subclusters both showed blueberry fruit sprayed with EBL exhibited higher relative abundance in flavone and flavanol subclusters. This may exhibit that EBL promotes the expression of FNS and LAR more than that of FLS and ANS in the flavonoid synthesis pathway of blueberry fruit. Li et al. (14) also found that spraying EBL increased the expression level of LAR while decreasing the expression level of FLS in grapes. This may lead to similar results as in this study, with an increase in the content of the flavonol subcluster and a decrease in the content of the flavonol subcluster in fruit. In the research on red-fleshed apples, Su et al. (11) found that MADS-box transcription factors in the brassinosteroid signaling pathway inhibited the expression of ANS, which can promote more substrate conversion to anthocyanins. In addition, compared to MT and MeJA, EBL significantly increased some notable compounds such as quercetin, leucocyanidin, phloretin, and epicatechin in blueberry fruit. Quercetin and epicatechin are recognized bioactive ingredients in blueberry fruit (14), and their increased contents are conducive to improving the health effects of blueberry fruit. Naringenin chalcone and phloretin, belonging to the chalcones subcluster, have been extensively investigated for their antioxidative, anti-inflammatory, anti-allergic, and anticancer properties, demonstrating potential health benefits (53, 54). Overall, while the impact of EBL on flavonoid accumulation in blueberry fruit may not be as significant as that of MT, it surpassed the impact of MeJA in this study. EBL may be better suited for fruits with a high demand for flavone and flavanol contents.

In this study, there were more differential flavonoid metabolites in MeJA vs. control, especially downregulated substances, which was inconsistent with TPC, TFC, and TAC results. This may be due to the relatively crude test methods used in TPC, TFC, and TAC or the limited number of widely targeted metabolome techniques. Overall, MeJA did not demonstrate significant superiority in enhancing flavonoid accumulation in blueberry fruit compared to MT and EBL. Notably, it was found that MeJA was more favorable for converting quercetin into downstream metabolites laricitrin and syringetin than MT and EBL. This may be because O-methyltransferases (OMT) play a key role in the synthesis of lari citrin and syringetin (55), and exogenous MeJA has been shown to significantly induce the expression of OMT in plants (33).

It has been confirmed that flavonoids are regulated by synthetic pathway enzymes (5). Plant growth regulators can influence these enzymes through their own signaling pathways or stimulate the production of other endogenous hormones to collectively regulate them through interactions (7). This results in the formation of a complex regulatory network, leading to differences between different plant growth regulators. Moreover, although there have been studies comparing the effects of different doses of plant growth regulators on fruit flavonoids, there is currently a lack of in-depth analysis regarding the reason. In this study, these enzyme activities may be responsible for the differences in the accumulation of phenolic compounds of blueberry fruit promoted by different plant growth regulators and different doses. However, further investigation is needed to elucidate the mechanisms by which MT, EBL, and MeJA affect these blueberry fruit enzymes, especially their impact on the enzyme gene expression. Although the regulation of plant growth regulators on the flavonoid synthesis of fruit is species-specific and tissue-specific (7, 21), we found some commonalities among the different species. Based on the aforementioned findings, it may be feasible to promote the accumulation of flavones and flavanols in fruits by spraying EBL and promote the accumulation of flavonols and anthocyanins in fruits by spraying MT. Therefore, EBL might be more appropriate for application in fruits that have high contents of flavones and flavanols, such as pear, mandarin, grapefruit, and mangosteen (45). MT might be more suitable for application in fruits with high levels of flavonols and anthocyanins, like blueberries and grapes (45).

## 5 Conclusion

In this study, MT, EBL, and MeJA promoted the accumulation of flavonoids without significantly accelerating the ripening of blueberry fruit at the experimental doses. Among them, 0.5 mM MT demonstrated the most effective promotion. The results revealed that MT, EBL, and MeJA had different effects on flavonoid subclusters in blueberry fruit. MT was particularly effective in increasing the contents of flavonols, anthocyanins, and flavanones, while EBL was more beneficial for increasing the contents of flavones and flavanols in blueberry fruit. However, MeJA did not show significant advantages in flavonoid accumulation compared to MT and EBL. Given that anthocyanins are a key indicator of the nutritional quality of blueberry fruit, MT appears to be the most suitable for producing high-quality blueberry fruit. However, further studies are needed to determine the optimal timing, frequency, and dose of MT application, as well as to investigate the mechanisms by which anthocyanins accumulate in blueberry fruit.

Although plant growth regulators exhibit species-specific characteristics, the effects of MT, EBL, and MeJA on flavonoid subclusters in blueberry fruit were similar to those observed in certain fruits. Plant growth regulators play distinct roles in regulating the accumulation of flavonoid subclusters in fruits. Therefore, the targeted and judicious selection of plant growth regulators based on specific flavonoid subclusters may be more effective in their application for producing high-quality fruits.

## Data Availability

The original contributions presented in the study are included in the article/supplementary material. Further inquiries can be directed to the corresponding author.

## Footnotes

^1^ https://www.r-project.org/
